# Cognitive and Behavioral Consequences of Sleep Disordered Breathing in Children

**DOI:** 10.3390/medsci5040030

**Published:** 2017-12-01

**Authors:** Irina Trosman, Samuel J. Trosman

**Affiliations:** 1 Sleep Medicine Center, Ann and Robert H. Lurie Children’s Hospital of Chicago, Chicago, IL 60611, USA; 2 Head and Neck Institute, Cleveland Clinic Foundation, Cleveland, OH 44195, USA; samuel.trosman@gmail.com

**Keywords:** sleep disordered breathing, obstructive sleep apnea, children, attention, learning, behavior

## Abstract

There is now a plethora of evidence that children with sleep disordered breathing (SDB) show deficits in neurocognitive performance, behavioral impairments, and school performance. The following review will focus on the neurobehavioral impacts of SDB, pediatric sleep investigation challenges, potential mechanisms of behavioral and cognitive deficits in children with SDB, and the impact of SDB treatment.

## 1. Brief Overview of Pediatric Sleep Disordered Breathing

Sleep disordered breathing (SDB) is a term used to describe a spectrum of breathing disorders during sleep. It includes, but is not limited to, habitual snoring, obstructive sleep apnea, and sleep-related hypoventilation [1]. This review will mostly focus on primary snoring and pediatric obstructive sleep apnea. 

Common nighttime symptoms of SDB include snoring, mouth breathing, apneas, gasping, labored or paradoxical breathing, excessive sweating, restless sleep, leg kicking, and hyperextension of the neck. Daytime symptoms may include inattentiveness, difficulty focusing, behavioral and mood problems, morning headaches, fatigue, excessive daytime sleepiness (EDS), and, in severe cases, failure to thrive. Persistent mouth breathing during sleep may be associated with dry mouth and complaints of thirst upon awakening. 

Primary snoring (PS) is defined as snoring without associated apneas, hypopneas, hypoxemia, hypercapnia, or sleep fragmentation [2]. There is no universally accepted, clear definition of snoring. Thus, the prevalence of the condition may differ based on varying perceptions of the word’s meaning across cultures. The overall prevalence of parent-reported snoring in one meta-analysis was 7.45% [3].

Obstructive Sleep Apnea (OSA) is characterized by recurrent episodes of upper airway obstruction during sleep that causes arousals, intermittent hypoxemia, and disruption of normal ventilation. According to International Classification of Sleep Disorder (ICSD)-3 criteria, OSA diagnosis requires the presence of at least one symptom (snoring, labored/obstructed breathing, or daytime consequences, such as sleepiness, hyperactivity) to be present, along with the sleep study findings [4,5]. 

Children with SDB may experience partial or complete obstruction of the upper airway during sleep. Recurrent episodes of airway narrowing or airway obstruction, associated with arousals or awakenings, disrupt sleep continuity. Sleep fragmentation and disrupted sleep architecture may result in nonrestorative sleep. Polysomnographic findings may include an elevated arousal index, decreased sleep efficiency, elevation of light sleep (stage N1), and reduction in overall amount of deep or Rapid eye movement (REM) sleep (stages N3 and REM) [6].

Up to 4% of typically developing children have obstructive sleep apnea, based on polysomnography (PSG) and approximately 11% have habitual snoring, based on parental report [3]. However, it is important to emphasize that the reported prevalence of SDB varies widely depending on the SDB definition and diagnostic methods deployed by investigators. For instance, the prevalence of SDB in some studies was estimated based on parental reports. However, parental reporting of snoring may not be reliable. Reporting depends on the frequency of child’s co-sleeping with the parents, and co-sleeping prevalence clearly differs across cultures [7]. Other studies collected data from diagnostic testing, such as nocturnal sleep laboratory based PSG [3]. Most studies consistently report a SDB peak between 2 and 8 years of age [6,8,9,10,11,12]. 

The current gold standard for the diagnosis of OSA, as recommended by the American Academy of Pediatrics (AAP), is a PSG study [9]. The cost of the procedure and lack of accessibility may preclude patients from having an overnight sleep study. An unfamiliar environment and PSG monitoring equipment could cause sleep disruption, making sleep study interpretation a challenge. 

OSA severity is determined by the frequency of obstructive apneas and hypopneas recorded on PSG. The apnea hypopnea index, or AHI, represents the average number of obstructive or partially obstructive events per hour of sleep. However, gas exchange abnormalities, such as hypoxia and hypercapnia, are frequently used as additional indicators of OSA severity.

Polysomnographic diagnostic criteria for OSA among adults and children are typically the product of expert consensus [13]. The AHI diagnostic criteria in children, when compared to adults, suffers from less available data and perhaps more heterogeneity across studies [3]. Part of the problem is that few studies have been performed to link specific levels of pediatric OSA with adverse outcomes. At present, an AHI of 1 to 5 events per hour of sleep, 5–10 per hour of sleep, and more than 10 events per hour of sleep is most often used to categorize mild, moderate, and severe OSA, respectively. However, various AHI definitions have been used in pediatric studies in the past. 

Although PSG provides an objective measure of sleep disturbance, measures derived from sleep studies are often not predictive of OSA-associated morbidities [14]. 

## 2. Challenges of Pediatric Sleep Investigations Focusing on Neurobehavioral Outcomes of Sleep Disordered Breathing

Various sleep disorders may result in sleep deprivation, sleep fragmentation, EDS, and symptoms related to increases in excessive sleep pressure. These disorders may have similar impacts on mood, attention, cognition, and behavior; however, SDB has been most extensively studied in this regard. 

Sleep fragmentation (resulting from respiratory related repeated arousals or brief awakenings) has been speculated to be one of the main causes of the neurobehavioral effects associated with SDB. However, changes in sleep architecture detected during PSG may be related to other co-morbid conditions, for instance “first night effect”. This latest phenomenon is attributed to the use of numerous electrodes and wires during PSG studies and an unfamiliar sleep environment. The first night effect frequently distorts sleep on the first night of recording. To mitigate this, sleep centers ideally should resort to using two or three nights of PSG recordings and discard the data from the first night. However, this is frequently not feasible [15]. 

The most definitive demonstration of treatment consequences in severe SDB would involve a double-blind, placebo-controlled, randomized trial. This study design is very difficult for several reasons. It is impossible to withhold treatment for severe cases of SDB for ethical reasons. In addition, families and clinicians cannot be blinded to surgical intervention (i.e., adenotonsillectomy) or sham surgery.

As mentioned above, the description of SDB prevalence is fraught with difficulty due to a variety of methodological issues. Some other challenges are listed below.

- Heterogeneity in OSA severity diagnostic criteria and lack of universally accepted specific pediatric polysomnographic parameters to distinguish primary snoring from OSA [3].
- The American Academy of Sleep Medicine (AASM) in 2012 modified the obstructive hypopnea definition, making it somewhat difficult to compare results from relatively recent studies with previously published papers [16].
- SDB is most prevalent in the preschool years (3–5 years of age); however, most pediatric studies focused on school-aged children [17,18]. Sleep fragmentation, disrupted sleep architecture, hypoxia, and hypercapnia are the presumed causative mechanisms for poor functional outcomes in SDB. They may be particularly important in younger children who are most susceptible to SDB related sequelae, due to particularly rapid cerebral and functional development [19,20].
- The patient population in studies may range from general community populations to children at tertiary care institutions (TCI) or subspecialty care with variable SDB severity. This may potentially create a referral bias.
- There are difficulties in evaluating behavioral problems in children of various ages by uniform tests. Younger children may not be able to verbalize excessive daytime sleepiness or fatigue. Their manifestation of disruptive sleep or excessive daytime sleep pressure may manifest as hyperactivity, impulsivity, increased aggression or oppositional behaviors. On the contrary, excessive sleep pressure in older children may be more similar to adults and present as excessive napping, yawning, rubbing eyes, complaints of fatigue or sleepiness, irritability, etc.
- Pediatric studies frequently rely on caregiver and teacher reporting of children’s behavior, attention, and hyperactivity levels as well as their estimated amount of sleep and sleep quality. These reports can be biased and affected by the caregivers’ cultural preferences, educational levels, expectations, and socio-economic backgrounds.
- A variety of potentially confounding factors increases the risk of cognitive dysfunction in children with any severity of OSA, such as premature birth, lower socioeconomic status, asthma, obesity, short sleep duration, and African-American ethnicity.
- Other factors may also have a significant impact on child sleep quality, alertness level, behavior, and development, such as other sleep disorders (i.e., circadian sleep disorders, restless leg syndrome, behaviorally induced insufficiency and/or disrupted sleep, parasomnias), various medical conditions (allergies, asthma, eczema, obesity, recurrent pharyngitis, etc.), medication use (i.e., use of nasal or inhaled steroids or anti-histamines, stimulant medications), genetics, and environmental factors (such as parental smoking).

Sorting through all of these issues is a tremendously difficult task. These challenges create substantial difficulties in interpreting research results and drawing meaningful conclusions.

## 3. Neurobehavioral Morbidity and Sleep Disordered Breathing

One of the earliest studies to shed light on the potential causative link between OSA and its detrimental consequences on academic performance was published in 1998 [21]. A remarkable increase in the prevalence of OSA was found in first-graders whose school performance was in the lowest tenth percentile of their class. Furthermore, the children who were treated for OSA showed significant academic improvements in their school grades in subsequent years, as opposed to untreated children. Another study reported that children who snored during early childhood were at greater risk for poor academic performance in later years, long after snoring had resolved [22]. 

Since then, there have been numerous mostly cross-sectional studies reporting the association between OSA and neurocognitive and behavioral morbidity. Many, albeit not all, studies have shown improvements in some of these functions after OSA treatment [23,24,25,26,27,28,29,30]. 

### Sleep Disordered Breathingand Excessive Daytime Sleepiness

The exact prevalence of excessive daytime sleepiness (EDS) in pediatric OSA is unclear. In contrast to adults, EDS does not tend to be a prominent complaint in children with SDB. EDS estimation in younger children is challenging and frequently relies on caretakers’ perceptions. Adults are frequently used as surrogate reporters because children are unlikely to verbalize their symptoms. It is also important to note that behavioral sleepiness may manifest differently in children than it does in adults. For example, children exhibit hyperactivity, inattentiveness, irritability, and/or oppositional behavior, rather than taking naps or having unintentional sleep episodes [31,32].

Several studies have examined the association between SDB and sleepiness in children by analyzing parental subjective reports. As opposed to adults with OSA, children were surprisingly relatively “protected” from OSA-induced hypersomnolence [33]. Some studies used objective measurements of EDS, such as the Multiple Sleep Latency Test (MSLT). The MSLT involves a series of brief daytime ‘nap’ opportunities in a comfortable sleep-conducive environment. The degree of sleepiness or “sleep pressure” is estimated by the child’s ability to fall asleep during naps. The shorter the latency to sleep onset during these nap opportunities, the higher the degree of sleepiness [34]. Unfortunately, MSLT assessments are expensive and difficult to conduct in children. In addition, MSLT has not been validated for younger children and there is a limited relationship between the sleepiness derived from a MSLT and the sleepiness based on subjective reporting [35,36].

Due to lack of objective measurements of sleepiness in younger children, it is not surprising that there is a paucity of pediatric studies objectively measuring EDS in children. Based on the available limited MSLT results, it appears that EDS in children with OSA is relatively infrequent and tends to be more noticeable among those with more severe OSA and/or obese patients [35,36,37]. 

### Sleep Disordered Breathing and Cognitive Function, Learning, and School Performance

Cognitive function is a mental act or process of knowledge acquisition. This process includes awareness, perception, intuition, and reasoning. Executive function encompasses the mental processes that enable children to plan, focus, remember instructions, and juggle multiple tasks successfully. Executive function is a domain that has been shown to be sensitive to intermittent hypoxemia, related to the obstructive sleep apnea syndrome. Thus, pediatric researchers focus on executive function to evaluate OSA related cognitive sequelae.

Executive function and self-regulation skills depend on working memory, mental flexibility, and self-control. 

The term working memory is often used interchangeable with short-term memory. However, short-term memory is just one component of working memory. Working memory requires temporary storage of information as well as the ability to accurately manipulate this information—for instance, baking a cake by memorizing ingredients and steps without making the unfortunate mistake of addition the same ingredient twice. Working memory has been found to correlate with intellectual function better than short-term memory [38,39,40,41]. Adult research has demonstrated a link between OSA and deficits of executive function [42]. Pediatric researchers also frequently use working memory performance and executive function assessment to evaluate neurocognitive deficits inflicted by OSA.

Impairments in executive functioning have been reported in multiple studies, including the Tucson Children’s Assessment of Sleep Apnea (Tuscan). The Tuscan study identified a negative correlation between AHI and immediate recall, Full Scale intelligence quotience (IQ), Performance IQ, and math achievement, while nocturnal hypoxemia adversely affected nonverbal skills [43,44]. 

Objective measurements of specific neuropsychological performance deficits related to sleep problems in children have been limited; however, pediatric studies have begun to identify significant differences in cognitive function between children with SDB and healthy controls. 

Verbal working memory impairments associated with OSA may compromise a child’s learning potential and neurocognitive development. For example, one study found significantly lower performance on measures of memory, executive function, and general intelligence in 5-year-old children with symptoms of SDB when compared to asymptomatic children [45]. Another study demonstrated decreased general intelligence, language, and visual-spatial skills in habitually snoring children [46]. The degree of neuropsychological deficits in children correlated with OSA severity [47]. In another study [48], working memory and basic attention tasks were evaluated in OSA children aged 8–12 years and compared to well-matched healthy controls. Sleep study findings and working memory functions were compared between the two groups. Compared to controls, children with OSA had poorer performance on both tasks of basic storage and central executive components in the verbal domain of working memory; however, no significant differences were detected in the visuospatial domain. The findings also suggested that AHI and oxygen saturation (SpO_2_) nadir were associated with verbal working memory performance. 

There is no consistency regarding the relationship between OSA severity and memory performance in children. While some studies revealed memory performance on standardized psychometric tests in children with OSA was significantly impaired, compared to healthy controls [49], with higher respiratory disturbance indices, correlating with greater memory deficits [50], other studies found no evidence of any differences in memory performance in children with varying degrees of OSA severity, when compared to control children [46,47,51]. 

School problems have been reported in multiple case-series of children with OSA [21,22,32], and such findings may underscore more extensive behavioral disturbances, such as restlessness, aggressive behavior, excessive daytime sleepiness, and poor test performances. 

### Sleep Disordered Breathing and Aggressive Behavior, Hyperactivity and Attention Deficit Hyperactivity Disorder

Multiple pediatric studies, based on parent or teacher surveys have found an association between childhood sleep-disordered breathing (SDB) and aggressive behavior, impulsivity, hyperactivity, and decreased attention [52,53,54,55,56,57,58].

For instance, one study demonstrated that in children with objectively confirmed SDB, aggressive behaviors are more frequent than in children without SDB, even when the SDB is mild [59,60]. Behavioral problems associated with sleep disordered breathing in school-aged children were also reported in the Tuscon study [60]. Another study focused on parental reporting of aggressive behavior among elementary school children. It demonstrated that aggressive children were twice as likely to have SDB symptoms compared to non-aggressive peers [61]

Multiple studies support the relationship between SBD and hyperactive behavior and inattentiveness [62]. SDB-related sleep fragmentation is speculated to be the cause of excessive daytime sleepiness, which may in turn interfere with sustained attention. Children with PS suffer from more sleepiness, inattention, and hyperactivity than healthy controls [63,64]. Despite the relative abundance of research in this area, there remain many unanswered questions regarding the relationship between sleep disturbances and attention deficit hyperactivity disorder (ADHD), and between OSA and ADHD-like symptoms in particular. The variations in diagnostic criteria for ADHD over the years (i.e., using different versions of Diagnostic and Statistical Manual of Mental Disorders criteria), the source of informant (e.g., parent, teacher, or clinical), and the reliability of rating scales used to diagnose ADHD, make the ADHD diagnosis difficult in some instances.

Interestingly, although children with ADHD appear to exhibit more sleep disturbances and symptoms of SDB than normal children [65,66] according to parental reports, only 20% of children with ADHD actually exhibited objective sleep disturbances when assessed by polysomnographic criteria [22,67]. In this study, it was also concluded that OSA is not more likely to occur among children with true ADHD when the ADHD diagnosis was established using the stringent criteria recommended by the Academy of Pediatrics and the Academy of Psychiatry.

Most studies demonstrate that parental questionnaire answers and ratings are sensitive to the behavioral aspects of SDB. Most parental ratings show that children with SDB are more symptomatic than control groups [23,68]. 

It is currently unclear whether sleep disturbances are intrinsic to ADHD, whether they are secondary to a co-morbid sleep disorder, or whether sleep disorders cause ADHD-like symptoms and thus result in a misdiagnosis.

In addition, some studies have shown that both children and adults with ADHD may exhibit symptoms of other sleep disorders, such as periodic limb movements of sleep, restless leg syndrome, delayed sleep phase syndrome, and initiation and maintenance insomnia [69,70,71,72,73]. 

A meta-analysis, analyzing eighteen studies published prior to 2012, was conducted to assess (1) the relationship between SDB and ADHD symptoms and (2) the extent of change in ADHD symptoms before and after adenotonsillectomy. The findings of this meta-analysis suggested that ADHD symptoms were related to SDB and improved after adenotonsillectomy [74]. However, the recent Childhood Adenotonsillectomy Trial (CHAT) study revealed no significant improvement in attention or executive function in children with non-severe OSA after adenotonsillectomy, as measured by neuropsychological testing [23]. 

### Sleep Disordered Breathing and Depressive Symptoms

Childhood depression is a significant problem that may lead to psychosocial problems and physical disabilities. Adolescents with depression have an increased risk of suicide and substance abuse [75].

Some studies have reported a high rate of depressive and anxiety symptoms (24%) among adults with OSA [76]. However, the relationship between depressive symptoms and OSA was no longer significant after controlling for age, body mass index (BMI), and hypertension [77]. Similarly, inconsistencies regarding the relationship between depression and OSA can be found in the pediatric literature. This may be attributed to methodological differences in OSA diagnostic criteria and evaluation of depressive symptoms. OSA related sleep disturbances and impairments in daily functioning may interfere with a child’s relationships with family, school, and peers [78]. 

A meta-analysis, designed to assess the relationship between depressive symptoms and OSA, and the efficacy of adenotonsillectomy for decreasing depressive symptoms reported a higher incidence of depressive symptoms among children with OSA, especially in males. OSA treatment with adenotonsillectomy was associated with decreased depressive symptoms when compared to pre-surgery levels. Thus, OSA screening in children and adolescents with depressive symptoms was suggested [79]. 

## 4. Are Cognitive Outcomes of Pediatric Sleep Disordered Breathing Reversed by Treatment?

Improvements in behavior, following treatment for OSA in children, were reported earlier [21,53] and suggested that at least some of the deficits may be reversible.

SDB therapeutic options include surgical interventions, with extraction of hypertrophic adenoids and tonsils being the most commonly performed procedures, as well as nonsurgical alternatives, such as watchful waiting, anti-inflammatory agents, orthodontic interventions, and positive airway pressure therapy (continuous positive airway pressure, also known as CPAP, or bi-level positive airway pressure, also known as Bi-LEVEL or Bi-PAP). 

Adenotonsillectomy is considered to be the first-line treatment in children [2,80]; however, not all patients are surgical candidates and some patients will continue to have symptoms after surgery [81,82]. Other surgical interventions, such as tongue-base suspension, uvulopharyngopalatoplasty, craniofacial surgery, bariatric surgery, and in most severe cases, tracheostomy, are used in selective pediatric populations [79]. 

### Effects of Adenotonsillectomy

Cognitive and behavioral abnormalities have been shown to be reduced after adenotonsillectomy (AT) in some [22,46,66,82,83,84], but not all [85], non-randomized studies, with inconsistencies in the reported effects after treatment. Previous studies have been limited by small samples, lack of randomization or appropriate controls, heterogeneous study groups, and frequent reliance on parent questionnaires, rather than on neuropsychological testing [66,84,86]. 

CHAT was the first large randomized controlled trial conducted to evaluate the efficacy of early adenotonsillectomy (eAT) versus watchful waiting with supportive care (WWSC) in children with OSA, with respect to cognitive, behavioral, quality-of-life, and sleep factors [23]. The primary outcome was a neurobehavioral measure of attention and executive function. Children 5 to 9 years of age with OSA without significant gas exchange abnormalities were randomly assigned to either an eAT group or a strategy of WWSC. They were evaluated at baseline and 7 months later. The primary outcome was evaluated by Developmental Neuropsychological Assessment (NEPSY), a test that has well-established psychometric properties [87]. 

Secondary outcomes included caregiver and teacher ratings of behavior, symptoms of the obstructive sleep apnea syndrome, sleepiness, global quality of life, and disease-specific quality of life.

The results revealed no correlation between the severity of the obstructive sleep apnea syndrome and treatment, with respect to the primary outcomes (attention and executive-function). However, eAT was found to reduce OSA symptoms and improve secondary outcomes (behavior, quality of life, and polysomnographic findings) [23]. In addition, the study secondary analysis revealed improvement in a time measure of selective attention and visual scanning in eAT group [88]. Interestingly, PSG of almost half of WWSC children had normalized by seven months. This finding suggested that non-severe OSA in school-age children may recover over time, without surgical intervention [89].

### Positive Airway Pressure Therapy

There are few published studies examining functional outcomes, such as neurocognitive outcomes, of children treated with positive airway pressure (PAP). An early study found that children with OSA who used PAP for 6 months had less daytime sleepiness, but no significant improvements in behavior, temperament or school performance. This study did not specifically assess attention, nor did it differentiate participants who were adherent to treatment from those who were not. It also used a school performance measure that was of unproven sensitivity to OSA. These methodological factors may have obscured changes in functioning for those individuals who were more adherent to PAP [90]. Another small outcomes analysis looked at self-reported academic grades, self- and parent-reported academic quality of life, and objectively-measured attention scores. These were assessed before and after PAP treatment in 13 obese adolescents with OSA as well as 15 untreated obese controls without OSA. The study demonstrated that non-adherent participants showed worsening functioning over time, while PAP users showed stable or improved functioning, similar to controls [91].

However, adherence with PAP therapy in adolescents with OSA is often a concern. Adolescents are notorious for diminished adherence to medical regimens and those who were prescribed PAP for OSA tended to follow this pattern as well [92].

In a study evaluating the effect of positive airway pressure therapy on neurobehavioral characteristics in a heterogeneous group of 52 children and adolescents, at baseline and after 3 months of treatment, PAP usage was associated with significant changes in neurobehavioral parameters, even in a heterogeneous group of children with OSA that included very young children and children with developmental delays [93].

Although it is difficult to get children to wear the PAP apparatus, it is important to encourage at least partial adherence to PAP therapy as those children who are even partially compliant with PAP therapy can display improved attention and academic functioning.

## 5. Potential Mechanisms of Neurobehavioral and Cognitive Dysfunction in Pediatric Sleep Disordered Breathing

The exact mechanisms by which OSA elicits neural deficits remain relatively unknown. Most likely, both the sleep fragmentation and episodic hypoxia associated with SDB lead directly to systemic inflammatory vascular changes in the brain [66,94,95]. Levels of inflammatory markers, such as C-reactive protein (CRP), interferon (IFN)-γ, tumor necrosis factor (TNF)-α and cytokine interleukin (IL)-6 have been shown to be elevated in children with SDB [96,97,98,99]. 

The findings linking vascular function and cognitive outcomes have been confirmed in several studies, such that the presence of endothelial dysfunction in OSA may serve as a surrogate reporter of altered cognitive functioning [100,101].

The frontal and hippocampal regions of the brain, which are implicated in the regulation of behavior and memory, respectively, appear to be most vulnerable to the effects of OSA. In children, gains in executive function skills occur during developmental periods, corresponding to the neuronal myelination and maturation of the prefrontal cortex [102,103]. Executive function is considered critical for school-age children, to develop complex problem solving [104] and to be able to perform other volitional tasks in response to new situations with demands on working memory [105]. If left untreated, OSA causes neuropsychological or executive dysfunction in developing children. If these skills are permanently impaired before maturation of the prefrontal cortex, this could severely alter a child’s cognitive potential, ultimately impacting both the child’s health and his or her functioning level in society.

In one study, researchers wanted to investigate the brains of children with OSA to see if there was any evidence of changes in the brain and if these changes were associated with any learning problems. They studied 31 children (19 with OSA and 12 healthy controls, aged 6–16 years). Participants underwent PSG and neuropsychological assessments, such as IQ tests and tests of their ability to perform tasks involving decision-making. Some of the children also had specialized scans of their brains (known as proton magnetic resonance spectroscopic imaging, or MRS) to measure the levels of certain metabolites produced as a result of brain activity. The researchers then compared the neuropsychological test scores with the levels of the metabolites. They found that relative to controls, children with severe OSA had lower IQs and a decreased ability to perform tasks involving decision-making. Children with OSA also had changes in metabolites in the brain, similar to those seen in diseases in which there is damage to brain cells. Compared to controls, children with OSA scored significantly lower on tests of overall intelligence and some aspects of higher-level thinking called “executive functions,” but the groups did not differ on tests involving sustained attention, motor skills, or visuospatial skills. Tests of memory did not yield significant differences between the groups, but the effect sizes were large enough for the authors to suggest that significant effects may have been found in a larger sample. MRS indicated that those with OSA had abnormal metabolites in the left hippocampus and right frontal cortex [85].

Additional factors, such as hypercarbia, architectural disturbances, including REM latency prolongation or reduction in REM sleep percentage as well as alternations in other stages of sleep may also play important roles.

It is important to emphasize that not all children with OSA exhibit cognitive deficits or behavioral problems. It has been speculated that both genetic and environmental factors may play roles in making some children more susceptible to the neurocognitive complications of OSA. Several genetic factors have been identified so far, including polymorphism within the NOX gene or its functional subunits [106]. The differences in systemic inflammatory responses, including plasma C-reactive protein (CRP) and interleukin levels may also be responsible for increased susceptibility to OSA related cognitive deficits [107]. Genetic variants of a gene critically involved in the formation of free radicals have been shown to account for discrepancies in cognitive outcomes in pediatric OSA [108]. Additional factors, such as insulin-like growth factor (IGF)-1 and apolipoprotein E allelic variants, have also been identified to be detrimental to neurocognitive function [108,109]. Some researchers have hypothesized that changes in regional cerebral blood flow during obstructive episodes leads to neurocognitive impairments [110].

## 6. Conclusions

There is now a plethora of evidence that children with SDB show deficits in neurocognitive performance, behavioral impairments, and decreased school performance; however, it is still not clear which factors (e.g., sleep fragmentation, degree of respiratory disturbance, hypoventilation, or oxygenation) play the most importance roles in neurocognitive morbidity. The exact mechanisms by which OSA causes neural deficits remain unresolved. 

Although sleep studies provide an objective evaluation of SDB severity, they are not predictive of OSA-associated morbidities. In addition, some children fulfilling current polysomnographic criteria for OSA do not manifest disease related morbidities. Other factors, such as individual susceptibility, co-morbid conditions, and environmental factors, appear to be important as well [14,111]. Some children with primary snoring display neurocognitive or cardiovascular sequelae and/or signs of systemic inflammation, despite normal sleep study findings [46,112]. 

Some SDB neurocognitive consequences are reversible while others may be irreversible if left untreated [22]. 

Despite some controversies, there is enough available data to support screening children with hyperactive behavior, inattentiveness, disruptive behavior, or learning disabilities for SDB and other sleep disorders. Screening for sleep disorders should be part of any well-child visit. Better education of pediatricians, general practitioners, mental health professionals, educators, and parents may help to identify and treat children at risk. An appropriate SDB diagnosis in children with ADHD symptoms may help prevent unnecessary stimulant use. Identification of the most severe SDB cases and subsequent early interventions are imperative to improve psychobehavioral short-term and long-term outcomes. Future research will help us better understand the pathophysiology of SDB and its effect on cognition and behavior in order to identify the most vulnerable children.
